# Among the Profession in New York State

**Published:** 1898-05

**Authors:** 


### AMONG THE PROFESSION IN NEW YORK STATE.

Dr. James L. Robertson, professor of theory and practice at the American Veterinary College, has been quite seriously ill for several months. Every veterinarian who has had the pleasure of forming the acquaintance of our genial colleague will wish for him a speedy and complete recovery. Dr. Robertson met with the loss of his father a few months ago.

Dr. James M. Richardson, recently house-surgeon of the New York College of Veterinary Surgeons, made a trip to England in March.

Dr. Herman Biggs has made an important communication on the subject of rabies to the New York City Board of Health.

Dr. Claude D. Morris, of Pawling, was a visitor to New York City in March, and purposes moving to Binghamton shortly.

Dr. R. S. Huidekoper, of New York City, is a great admirer of bull-terrier dogs, and in "Corona" had the prize-winner for several years in America.

Dr. H. F. Foote, of New Rochelle, finds much pleasure among his dogs, and his kennels contain many fine specimens among the toy varieties.

Dr. R. B. Plageman, of Brooklyn, loves the field of canine practice. He thinks rabies an extremely rare disease, and the dangers of dog-shows in spreading this affection not worth considering.

Dr. G. Howard Davison, of Altamont Stock-farm, owns some of the best Shropshire sheep in the world. At Millbrook he finds much pleasure and recreation in attaining the highest excellence among his flock.

Dr. William H. Pendry, of Brooklyn, was for many years a great admirer of the thoroughbred horse, until the days of the "dope" robbed them of much of their attraction.

Dr. William Sheppard, of Sheepshead Bay, says there is no form of animal that can compare in beauty, power, and feats of speed with the thoroughbred, and legislation destined to limit the sport of man from time immemorial is a step backward in the race of civilization.

Dr. J. Elmer Ryder, of New York City, finds the keenest delight mounted high on a skeleton brake-wagon behind a pair of high-stepping hackneys.

Dr. Richard E. Buckley, of the borough of Manhattan, finds a deal of pleasure along the speedway in testing the racing qualities of those who seek this spot to fly their racers.

Dr. W. D. Critcherson, of Greater New York, finds a pleasant family horse and the company of his wife and children on a quiet afternoon's drive in Central Park a great relief from the pressure of professional work.

Dr. L. McLean, of Brooklyn, finds the driving of horses irksome, and would rather use "shank's mare" in his perambulations around the borough of Brooklyn.

Dr. H. D. Gill, of New York City, cannot keep away from the sale-centres of trotters, and his fast steppers on the racetrack have won him fame and courted fortune.

Dr. E. B. Ackerman, of Brooklyn, spends all his spare hours in the "City of Churches" looking after the welfare of the horses of that city from inhuman drivers and worse owners.

Dr. J. S. Cattanach, of Greater New York, finds his keenest delight in following his professional duties behind a pair of trotting mules.

Dr. Nelson P. Hinkley, of Buffalo, takes pride in his pair of well-drilled horses and ambulance. Their prompt response to the gong and eager way of responding to duty increase their worth and attraction to the owner.

Dr. Benjamin P. Wende, of East Buffalo, thinks the attraction of a flock of sheep greater than any of the domesticated animals. He likes their gentle ways.

Dr. Claude D. Morris, of Binghamton, admires the animal that fills to overflowing the family pail and which furnishes the best of foods in all ages of man.

Dr. John Dougherty, of New York City, finds the heavy draught-horse the highest perfection of animal power and worth and his exhibition of unlimited strength in hauling great burdens a sight worthy to behold.

Dr. F. C. Grenside, of New York City, revels in the proper training and a perfectly made toilet of a horse prepared for the sale-mart.

Dr. S. S. Field, Jr., of New York City, considers the well-trained stage-horse the acme of perfection, and would rather follow the drilled stage-horses than pursue the uncertainties of routine practice.

Dr. Cooper Curtice, of Moravia, would rather fathom out the way and the methods of transmission of the infectious and contagious diseases of the domesticated animals than to be tied to the care of everyday cases of routine practitioners.

Dr. Herbert Neher, of New York City, would rather look through the lens of a microscope and view the field of nature's complete work than study the uncertainties of the firing-iron.

Dr. Olof Schwarzkopf, of Flushing, thinks that life is not worth living unless one can combine the field of teaching with that of daily investigation.

Dr. John Faust, of Poughkeepsie, says that student-life never ends in comparative science, and only mature years of study and investigation make one worthy of the name of veterinarian.

Dr. William H. Kelly, of Albany, spends many sleepless nights as to how one is to maintain the pristine attraction of State laws for regulating the practice of veterinary science, and keep them from being violated, or the discovery of loopholes and flaws for evading their requirements.

Dr. Arthur O'Shea, of New York City, is now out of an occupation. The end of the legislative session in the Empire State fills his heart with sadness, for there remain no other worlds for him to conquer. Conquest by legislation is the target of his powers.

Dr. R. W. Hickman, of New York City, wonders what foreign countries will find next to prohibit American meat-products, and what additional vigilance is possible to demand of him and his able staff in this part of the Government service.

Dr. H. Clay Glover, of Greater New York, says there can be no more attractive sight to man than a smart trap, a fetchingly costumed lady, and a well-fed and groomed dog spinning along the drives of Central Park.

Dr. Charles Cowie, of Ogdensburg, says that official duties in watching ports of entry that may be free from dangerous diseases is calculated to try thoroughly one's patience and judgment.

Dr. B. H. Pendry, of Brooklyn, has been called to military duty with the 13th Regiment of New York State.

Dr. John A. Bell, of Watertown, finds the greatest difficulties in veterinary sanitary police-work in educating public sentiment to a proper appreciation of the need and value of stringent measures in the production and sale of milk.

Dr. R. R. Bell, of the "City across the Bridge," says of all the thankless tasks one might be heir to, that of editorial fame outstrips them all. Its returns are wholly disproportionate to the time and labor given, and causes him to wish many times that some of his correspondents had his job for a season; they could do it much better.

Dr. H. S. Wende, of Tonawanda, a member of the 25th Separate Company of New York State Militia, has been called to the front in the Hispano-American war.

Dr. Edward N. Leavy, of Gotham, considers the domain of canine and feline practice the sphere of women veterinarians, and there will never be a proper regulation of matters veterinary until the college doors are open to students of the gentler sex.

Dr. Cooper Curtice, of Moravia, was a visitor to Philadelphia in April, and will engage in some special work in the Keystone State under the direction of the Pennsylvania Livestock Sanitary Board.

Dr. William H. Pendry, of Brooklyn, of Troop "C," National Guard of New York, has fitted up one of the most complete veterinary equipments in conjunction with any of the cavalry of New York State. Dr. Pendry is a member of Troop C and holds himself in readiness to respond to the nation's call should his services be required.

Dr. L. E. Willyoung, of Buffalo, has moved from 395 Ellicott Street to 119 West Tupper Street, and is making a specialty in the treatment of dogs.

The Bull-terrier Club of America, of which Dr. R. S. Huidekoper is secretary-treasurer, had their inaugural bench-show of all terriers and bulldogs at the American Horse Exchange, April 21st and 22d. Over three hundred dogs were benched, and the show as a whole was very successful. Dr. Huidekoper was superintendent and Dr. H. D. Gill was veterinarian.

Dr. Gill was very pleasantly entertained by Dr. and Mrs. Bell at their very pretty home in Brooklyn. Messrs. Gill and Bell discussed veterinary education and the veterinary colleges until the small hours of the morning.

Drs. Eli Strauss and Alphonse J. Dodin have been appointed veterinary inspectors of the Health Department for the Borough of Bronx.

The following is a list of veterinarians now in the Government employ in the district of Greater New York: Drs. William H. Rose, inspector-in-charge of export live-cattle; H. Brister, R. R. Letts, R. W. Hickman, inspector-in-charge, Wilbur J. Murphy, Louis Abel, E. A. Parsons, James H. Ferster, John B. Hopper, and W. J. Reagan.

Dr. R. S. Huidekoper has been called to Washington on military matters.

Dr. Claude Morris, while in New York recently, visited the American Veterinary College and the New York College of Veterinary Surgeons.

Dr. Thomas Giffen, who has been suffering from locomotor ataxia for some time, has permanently retired from business. Dr. Blakesly succeeds him in practice.

Francis D. Bowne, senior student of the New York College of Veterinary Surgeons, has gone to the front as Quartermaster-Sergeant of Squadron A.

One of the editors of the JOURNAL had the pleasure of inspecting Dr. R. R. Bell's hospital in Brooklyn. His office had an air of methodical business prosperity, with a well-stocked pharmacy and instrument-case, in which were noticed all of the latest improvements in tools. The hospital is fully equipped with soaking-stall, an excellent set of stocks and a most commodious box-stall for colic cases or paddock. His buggy, and that of his assistant, Dr. Jenks, are equipped with an emergency kit—mixtures of all sorts—tooth-rasps, firing-irons, etc.—and, in fact, the most perfect outfit ever seen by the writer.

Dr. Herbert Neher, of New York City, has severed his connection with the Metropolitan Traction Company, where he has been for the past ten years, and is about to establish himself permanently at Fort Edward, Washington County, N. Y., where he intends to build himself a substantial home and hospital. As the doctor's health has not been the best for the past year or more, he hopes to derive benefit by the change.

Dr. George P. Biggs, Secretary of the New York College of Veterinary Surgeons and Professor of Physiology, has just returned from his wedding tour.

Dr. J. C. Cattanach, Jr., is about to start for the Klondike.

Dr. Veranus A. Moore, of the New York State Veterinary College, has made a number of valuable contributions to the study of veterinary pathology during the past year.

Dr. John J. Cattanach, of New York City, will be one of a prospecting party to enter the Klondike region about April 1st.

Dr. H. D. Hanson, of New York City, will shortly issue two books of interest and importance to the veterinary profession.

Veterinarian G. Howard Davison was a visitor to Detroit, Mich., in February.

Drs. T. G. Sherwood and H. Clay Glover were the veterinarians to the Westminster Kennel Club Show at Madison Square Garden in February.

Without a vacant place and with one of the best dinners ever spread, surely the kind host and hostess, Dr. and Mrs. H. D. Gill, of New York, must have enjoyed the evidences of appreciation on all sides exhibited by their guests on March 3d, Drs. Austin Peters, Leonard Pearson, Wm. H. Lowe, W. Horace Hoskins, E. B. Ackerman, R. R. Bell, J. S. Cattanach, J. H. Ferster, R. W. Hickman, J. E. Ryder, with Drs. H. D. Gill and R. S. Huidekoper seated at the respective ends of the beautifully arranged and decorated table.

### THE EMPIRE STATE VETERINARY COLLEGES.

The New York College of Veterinary Surgeons; chartered in 1857; granting the degree (V.S.) Veterinary Surgeon; still in active existence, and located now at 154 E. 57th Street, New York City. Prof. H. D. Gill, Dean.

The American Veterinary College; chartered in 1875; granting the degree (D.V.S.) Doctor of Veterinary Surgery; still in active existence, and located at 141 W. 54th Street, New York City. Prof. A. Liautard, Dean.

The Veterinary Department of Cornell University was created at the inauguration of the University in 1868.

Since that time the right has been maintained to grant degrees in veterinary medicine.

The degrees granted were bachelor and doctor of veterinary medicine, the former being the graduate degree given on the basis of four years of successful study in the institution. The doctorate was an advanced degree granted after two more years of successful study, which might, however, be *in absentia.*

The Columbia Veterinary College; chartered in 1878 ; granted the degree (D.V.S.) Doctor of Veterinary Science; closed its doors 1884, by amalgamation with the American Veterinary College.

In the New York State Veterinary College, which has suspended the veterinary department of the University, the degree of doctor takes the place of the old University degree of bachelor, and is granted on the basis of three years of successful study in place of four, as formerly required in the University veterinary department.

The New York State Veterinary College is located at Ithaca, on the campus of Cornell University, and the Legislature has voted thousands of the people's money to equip it and keep it going. The announcement for the coming year gives the class for 1897–1898. There are five third-year students, only one of whom is from New York; eight second-year students, six of whom are from Ithaca ; and four first-year students. This is a very weak showing for the amonnt of money expended. What is the canse? The standard of admission to this and other veterinary colleges in the State has been made so high by the Board of Regents as to practically bar the majority of young men who aspire to the practice of veterinary medicine. A certificate of forty-eight academic counts is required to enter. The number of counts represented by each subject is : English, 8 ; geography, physical and political, 2 ; drawing, 2 ; American history and civics, 2 ; plane geometry, 4 ; algebra, 4 ; elementary French, 4 ; elementary German, 4 ; Latin, Cæsar and grammar, 8 ; chemistry, 4; and geology, 4. This is a formidable list, and it is pronounced unreasonable by some of the foremost scientific men in the State. If the Regents do not reduce the number of counts the Legislature should take the matter in hand next winter. It is absurd to spend the people's money in such a way as to furnish such insignificant results. While the veterinary colleges of this State are being strangled by the action of the Regents, those of other States and Canada are doing a flourishing business. Students unable to pass a preliminary examination here find no difficulty in entering the schools at Toronto, Montreal, and elsewhere.—*Turf, Field, and Farm.*

In March there was a conference of representatives of the three veterinary schools with the Board of Regents, and the Regents' counts were reduced from 48 to 24, or a two-year instead of a fouryear high-school course, and under the present ruling of the Board students can enter any of the New York veterinary schools with only twelve counts, and have until the beginning of their second session to make up the other twelve. The Regents also recommended a union of the two New York schools, in the event of which such a school might become connected with the Columbia University. At the Regents' suggestion, a committee was appointed by the Trustees of the New York College of Veterinary Surgeons to bring about such a union.

Melvill Dewey, Secretary of the Board of Regents, at the conference of Colleges, put himself on record by saying that New York City, on account of its clinical advantages, was the proper place for a veterinary school.

Cornell has acquired a medical department, to be established in New York City.

Amalgamation of the two New York veterinary schools and their affiliation with Columbia University, with a yearly endowment. The Faculties of the two schools would make an excellent teaching-staff. Such a union and affiliation would insure perpetuation of both schools and the names of the founders, Dr. Liautard in particular.

Reduction of Regents' counts from 48 to 24 in the Empire State for veterinary students.

---

### NEW YORK ASSOCIATIONS.

*New York State Veterinary Medical Society.* Officers : President, W. L. Baker, Courtland ; Vice-President, Roscoe R. Bell, Brooklyn ; Secretary-Treasurer, C. D. Morris, Binghamton. Meetings : Annually in September.

*Veterinary Medical Association of New York County.* Officers : President, R. S. Huidekoper, New York City ; Vice-President, James L. Robertson, New York City ; Secretary, Robert W. Ellis, New York City ; Treasurer, H. D. Hanson, New York City. Meetings : First Wednesday of each month.

*Niagara and Orleans County Veterinary Medical Association.* Officers : President, James Martin, Lockport ; Vice-President, W. E. Stocking, Medina ; Secretary-Treasurer, J. P. Thomson, Niagara Falls. Censors : George Kesler, Holly ; W. R. Hunter, Niagara Falls, and M. D. Williams, Middleport. Meetings : January, April, July, and October.

*Oneida County Veterinary Medical Association.* Officers : President, F. Morrow, Oneida ; Vice-President, Wilson Huff, Rome ; Secretary-Treasurer, J. M. Currie, Rome. Meetings : Quarterly.

*Genesee Valley Veterinary Medical Association.* Officers : President, Albert Drinkwater, Rochester ; Vice-President, W. G. Dodds, Canandaigua ; Secretary, Albert Tegg, Rochester ; Treasurer, L. R. Webber, Rochester. Meetings : Semi-annually, January and July.

*New York German Veterinary Medical Association.* Officers : President, G. R. Sattler, Newark, N. J.; Vice-President, Rudolph Leis, Newark, N. J.; Secretary, Edwin Ancker, New York City ; Librarian, Otto Leis, Newark, N. J. Meetings : Monthly.

---

### WHAT NEW YORK VETERINARIANS WANT.

The placing of the control and eradication of bovine tuberculosis in the hands of the veterinarians.

To know why nearly eight hundred devotees of this special science are still unable to have its own work directed by veterinarians?

Why so many municipal and district Boards of Health are without a veterinary representative?

To know why all the work in the State Legislative halls is placed on the shoulders of a few self-sacrificing members of the profession?

A legislative organization in 1898 comprised of an active member of the profession in every county of the State.

A keener interest in the work of the State veterinary organization by the members of the profession in Greater New York.

More fraternal spirit that will unite in a common determination to place its best men in the highest places.

A keener interest in their alma mater by the alumni of the Empire State.

A reduction in the preliminary education of veterinary students from forty-eight counts.

Rank in all the military bodies of the State.

Amalgamation of the veterinary colleges of New York City.

A veterinary college maintained in New York City where such great clinical material is to be obtained.

Some of her wealthy people to take a keener interest in veterinary education and promote the work in this direction by the endowment of veterinary chairs.

More attention to the needs of the colleges by their boards of trustees.

More county veterinary organizations.

More of their number to take a keener interest in veterinary journalism as contributors.

More liberal knowledge among laymen of the scope of the veterinarian's education.

A better conception of the responsibilities of veterinarians by those who employ them.

A higher standard of ethical relations, a closer fraternal spirit among veterinarians.

---

### WHAT A NEW YORK HOUSE HAS DONE.

THE centre of veterinary publications in America has been located for nearly twenty years in New York City, and the veterinary profession owe much to the liberality of the well-known publishing-house of William R. Jenkins. At all times this house has stood ready to add to the veterinary literature of our country all worthy offerings for the better education of our members. It still leads in this special publication field, and during the past twelve months has been unusually active in bringing forth a number of books of special merit and importance. Every veterinary publication of the world is to be obtained through this firm, and it is a distributing centre of great importance to the welfare and convenience of our votaries. We desire to make this public acknowledgment of our indebtedness as a profession to the generosity that has marked the career of the firm of William R. Jenkins and congratulate them on the excellent books they have brought forth so recently.

The subject of horseshoeing, of so much daily practical importance to the veterinarian, has recently been specially favored in the addition of no less than three worthy books on this all-important subject. Two of these have been placed at our command by this well-known firm. The *Art of Horseshoeing*, a manual for farriers, by William Hunting, F.R.C.V.S. A book of one hundred and twenty-six pages, with nearly one hundred illustrations. While a book primarily intended for the farrier, horseowner, and student of the art of shoeing, it is a very readable book for the advanced veterinarian, and presents in an interesting manner, in a series of twelve chapters, the form and action of the foot; preparation of foot for shoeing; foals and unshod feet; form and manufacture of shoes; selection of a shoe; fitting and application of shoes; on roughing; injuries resulting from shoeing; shoeing bad feet; leather and rubber pads; shoeing competitions. While, perhaps, no subject affords so wide a variance of opinion, and many will differ strongly with the author on many points, still few will read the book without feeling that it was time well spent, and that a better knowledge of this important subject had been obtained.

More complete and extended in all directions is *A Handbook of Horseshoeing*, with introductory chapters on the anatomy and physiology of the horse's foot, by John A. W. Dollar, M.R.C.V.S., with the collaboration of Albert Wheatly, F.R.C.V.S. The author has in its preparation consulted the writings of almost every one who has anything in print on this subject, and thus brought to his contribution the best thoughts on horseshoeing the world over. Part I. covers the structure and functions of the foot, and this is complete in every detail. In Part II., the horse's foot in relation to shoeing, every form and type of shoe, every principle sought to be followed, every type and special-purpose horse, and the many specially designed shoes for them in favor at home and abroad, are dealt with; the various lesions of the foot due to or to be remedied by shoeing are most thoroughly treated, and a volume of information brought to the subject that has not been attempted before by any English writer. The shoeing of oxen is added, with a chapter on the structure and functions of the ox's foot; also an appendix covering farriers' teaching-schools and shoeing competitions. It is a book approaching nearly five hundred pages, with over four hundred illustrations, and contains the most complete collection of illustrations of the various kinds and forms of shoes ever published. It is a book that will best be appreciated by those who will give time and careful study to its contents.

Through this same house, Prof. L. H. Freidburg, of the American Veterinary College, has presented a translation of Prof. Rudolph Kobert's *Practical Toxicology*, from the third German edition. This book is one of unusual merit, and student, veterinarian, and physician alike will appreciate its merits and value. Its tables, so graphically arranged, so ready of consultation, and so complete in advice, will prove invaluable and many times give instant and inestimable aid in those sudden emergency cases where poisoning is suspected and when one wants the most concise and complete assistance to be of avail. Its appreciation to the extent of a third edition in German within a few years is a fitting testimonial of its merits, and every English speaking and reading veterinarian will be thankful many times to Prof. Freidburg for his excellent translation. It is a book of more than two hundred pages, with a very complete alphabetical index, printed on excellent paper in clear, large-faced type.

W. H. Dalrymple, M.R.C.V.S., in presenting his compend on *Veterinary Obstetrics* gratefully dedicates the same to Prof. A. Liautard, of the American Veterinary College, as a token of appreciation of his labors in America for the veterinary profession. This book, of one hundred and sixty-two pages, with fifty-one illustrations, intended especially for student-life and a reference-book for the busy practitioner, will be found to fill a very acceptable place without in any way detracting from the more extended works at our command on this subject. Its convenient arrangement in chapters covering the various aspects of this subject adds to its value as a ready reference-book. The author's experience as a teacher in agricultural colleges has suggested its presentation typographically in an attractive form, and it will no doubt contribute very much to the more successful study of this subject in every school where veterinary obstetrics is taught.

*Breeding Race-horses by the Figure System*, compiled by the late C. Bruce Lowe and edited by William Allison, is one of those interesting contributions to the study of the progress of breeding, that, no matter how much one may differ with the deductions and views of the author, nevertheless has an interest and attraction of no little merit to every student of the genealogy of race-horses and the potency of certain animals in the transmission of certain qualities and power, the chief desiderata in the possession and pleasure of ownership of such animals. To make this more readily understood the author groups the great racing progenitors of the thoroughbred family, and numbers them in the relative estimate of their achievements, and thus follows every great horse and places him by numbers in the class his powers merit, thus more readily showing to what lines such animals owed their racing propensities. Why this prepotent power, and in what way it is transmitted, forms an interesting chapter of deductions, and can be read with profit by every horse-lover and admirer.

It is rare to note that one firm contributes so much to the betterment and progress of a vocation in so short a period of time as has been presented by this well-known firm, and specially so when the work of the veterinarian has been so much curtailed in its chief direction during the past two years; but it is fitting to note at this time the confidence thus expressed and the suggestion it carries that, on the whole, the veterinarian's education has not been broad enough on certain lines, and his greatest drawback has been the lack of proper text-books for his scientific advancement. We believe these contributions will be appreciated to such an extent as to give encouragement toward the production of others that will fill equally demanded needs of the veterinarian.

